# Supplementary material for: The Influence of Food Regimes on Oxidative Stress: A Permutation-Based Approach Using the NPC Test
Source: Healthcare (Basel). 2023 Aug 11;11(16):2263. doi: 10.3390/healthcare11162263 (PMC10454221; doi:10.3390/healthcare11162263)
Supplement: Supplementary file 1 [file healthcare-11-02263-s001.zip › healthcare-2502598-supplementary.pdf]

healthcare

Supplementary Materials

Data Collection

Participants to the study were recruited during educational campaigns for the general population, which were run at our Institution. On these occasions, we usually organize informational events, provide educational materials and offer free endocrine consults to people who voluntary join the campaign. We enrolled our study group among the adult participants to these information events. All participants were asked to complete a self-reporting questionnaire, that was administered at the first consult by a physician (the PI of the study), to collect data regarding lifestyle and dietary habits. We used the approach of a food frequency questionnaire, evaluating the intake frequencies of food groups (meat, fish, cereals, fruits and vegetables, dairy products) to assess dietary habits. For this purpose, we used a validated Italian questionnaire [1] with the help of a picture boocked to show portions. The questionnaire has been already developed and validated by a panel of experts from the Italian INRAN (*Istituto Nazionale di Ricerca per gli Alimenti e la Nutrizione*) Study Group and it has been widely used for national food consumption survey, to update dietary information in our country [2–5].

Using information from the diet questionnaire and the FFQ, participants were classified in two different diet groups: semi-vegetarian (consuming red meat, poultry or fish no more than once a week) and omnivorous (eating meat or fish almost more frequently), according to other studies in the literature [6]. No further subdivision (vegan, not consuming any animal products, or vegetarian not consuming any meat or fish) was done because of the small number of subjects under study.

Along with eating habits, the participants were asked to record: i. demographic (sex, date of birth, age at time of the present survey, educational background, employment, cohabitation, marital status,…) and anthropometric data (height, weight, BMI, …); ii. information on their lifestyle, including: smoking habits; alcohol consumption; eating habits (time and place when meals are usually consumed; specific dietary regimens such as gluten-free, lactose-free, vegan diets); physical activity; iii. Information on any previous disease, any drugs or supplements taken in the previous six months (for inclusion/exclusion criteria).

Physical activity (PA) was subdivided into two categories: leisure time PA (e.g., sports, recreation, and transportation), and occupational PA

Occupational PA was quantitated using the following question used in other studies [7,8]:

'What is your physical activity at work within the last year?'

- Predominantly sedentary work.
- Sedentary or standing, sometimes walking work.
- Walking, sometimes lifting work.
- Heavy manual work.

Leisure time physical activity was quantitated by the following question used in numerous previous studies [see references below]

'What is your physical activity during leisure time (including transport to and from work) within the last year?'

- Almost completely sedentary or light physical activity less than once a week (or less than 2 hours per week).
- Light physical activity for at least once a week (or 2–4 hours per week).
- Light physical activity for more than one a week (or more than 4 hours per week) or vigorous physical activity for at least once a week (or 2–4 hours per week).
- Vigorous physical activity or regular heavy exercise or competitive sports several times per week.

References

1. D'Addezio L, Capriotti M, Pettinelli A, Turrini A 2011 L'indagine nazionale sui consumi alimentari in Italia INRAN-SCAI 2005-06. Parte C: I risultati dei questionari sulle abitudini alimentari. Osservatorio Consumi Alimentari, INRAN. Roma
2. Istituto Nazionale di Statistica (2001) 14° Censimento Generale della Popolazione e delle Abitazioni del 2001. Rome: ISTAT; available at http://dawinci.istat.it/MD/Google Scholar.
3. Leclercq, C.; Berardi, D.; Sorbillo, M.R.; Lambe, J. Intake of saccharin, aspartame, acesulfame K and cyclamate in Italian teenagers: present levels and projections. *Food Addit. Contam.* **1999**, *16*, 99–109.
4. Leclercq, C.; Arcella, D.; Piccinelli, R.; Sette, S.; Le Donne, C.; Turrini, A. INRAN-SCAI 2005-06 Study Group. The Italian National Food Consumption Survey INRAN-SCAI 2005-06: main results in terms of food consumption. *Public Health Nutr.* **2009**, *12*, 2504–2532.
5. Leclercq, C.; Piccinelli, R.; Arcella, D.; Le Donne, C. Food consumption and nutrient intake in a sample of Italian secondary school students. Results from the INRAN-RM-2001 food survey. *Int. J. Food Sci. Nutr.* **2004**, *55*, 265–277.
6. Clarys, P.; Deliens, T.; Huybrechts, I.; Deriemaeker, P.; Vanaelst, B.; De Keyzer, W.; Hebbelinck, M.; Mullie, P. Comparison of nutritional quality of the vegan, vegetarian, semi-vegetarian, pesco-vegetarian and omnivorous diet. *Nutrients* **2014**, *6*, 1318–1332. doi: 10.3390/nu6031318.
7. Holtermann, A.; Marott, J.L.; Gyntelberg, F.; Søgaard, K.; Suadicani, P.; Mortensen, O.S.; Prescott, E.; Schnohr, P. Occupational and leisure time physical activity: risk of all-cause mortality and myocardial infarction in the Copenhagen City Heart Study. A prospective cohort study. *BMJ Open* **2012**, *2*, e000556.
8. Holtermann A; Schnohr, P.; Nordestgaard, B.G.; Marott, J.L. The physical activity paradox in cardiovascular disease and all-cause mortality: the contemporary Copenhagen General Population Study with 104,046 adults. *Eur. Heart J.* **2021**, *42*, 1499–1511.
